# Nutritional, Phytochemical, and *In Vitro* Antioxidant Activity Analysis of Different States of Soy Products

**DOI:** 10.1155/2022/9817999

**Published:** 2022-09-13

**Authors:** Rahat Bin Robbani, Md. Munnaf Hossen, Kanika Mitra, Md. Zahurul Haque, Md. Abu Zubair, Shumsuzzaman Khan, Md. Nazim Uddin

**Affiliations:** ^1^Department of Food Technology and Nutritional Science, Mawlana Bhashani Science and Technology University, Santosh, Tangail 1902, Bangladesh; ^2^Institute of Food Science and Technology (IFST), Bangladesh Council of Scientific and Industrial Research (BCSIR), Dhaka 1205, Bangladesh; ^3^Department of Biochemistry and Molecular Biology, Jahangirnagar University, Bangladesh

## Abstract

Consumer demand for food nutritional content and quality is driving the design of plant-based foods that are enhanced with proteins. In this study, we aimed to reveal the nutrient compositional differences of various states of soy flours. We compared soy protein concentrate (SPC) with full fat (FF), raw soy flour (RSF), and defatted (DF) soy flour for investigating nutritional content, phytochemicals, and *in vitro* antioxidant activity. The results showed that the SPC contained significantly (*p* < 0.001) higher protein content (65.14%) and low-fat content (0.54%) than RSF, FF, and DF. Furthermore, the findings revealed that all products contain a significant (ANOVA, *p* < 0.001) amount of essential minerals. The RSF contains significantly higher (*p* < 0.001) potassium (1178.6 mg), calcium (216.77 mg), and magnesium (247 mg) per 100 g than FF, DF, and SPC. SPC contains essential amino acids, but we were unable to detect phenylalanine and tryptophan due to a limitation in the method. Furthermore, using methanolic and aqueous extracts of RSF, FF, DF, and SPC, the flavonoid, phenolics, and antioxidant capacity were also evaluated. According to the findings, soy products in methanolic extract had higher phenolic (about 12-34 mg/g) and flavonoid (about 63-150 mg/g) levels than aqueous extract. Results also demonstrated that FF had higher phenolic content, and SPC had higher flavonoid content than the other products. *In vitro* models such as phosphomolybdenum blue, FRAP, DPPH, and ABTS assays were used to study the total antioxidant and free radical scavenging potential of soy products, and results found that soy products contained a significant (*p* < 0.001) amount of antioxidant equivalent to gallic acid and vitamin C standard. In the DPPH and ABTS assays, the results also showed that soy products can reduce free radicals in different *in vitro* models. Altogether, these findings suggest that soy flours, particularly DF and SPC, could be a beneficial food ingredient in the formulation of functional foods.

## 1. Introduction

Soybean (*Glycine max*) is a pea family member that grows well in tropical, subtropical, and temperate climates. It is also known as an oil seed, however, after processing, it is utilized as a food ingredient. Soybean contains the most protein and isoflavones of any leguminous plant, which may help to prevent protein deficiency as well as cancer and osteoporosis [1]. As soybean is richer in macronutrients such as high protein and fat than other legumes, it could be a potential food component for vegetarians [2, 3]. Despite being relatively new to American consumers, soy products have long been popular in Japan, China, and Korea [4]. Consuming soy products may lower the risk of type 2 diabetes due to their low glycemic index [5]. It contains isoflavones, which are necessary for the heart, kidneys, colon, liver, and stomach for normal functioning. According to a meta-analysis, soy protein is linked to significant reductions in blood cholesterol, low-density lipoprotein (LDL) cholesterol, and triglyceride levels [6].

Soybeans are becoming a popular food component around the world due to their high nutrient content. It is directly used for plant-based food products such as soy milk, tofu, natto, and soy sauce and is also used as a food ingredient (soy flour) for developing functional foods [7]. Soy flour, which is high in protein, has recently attracted attention for the development of flour-based functional foods. According to studies, processed flour has higher protein than raw soy flour (RSF) [8].

Through the acid leaching or aqueous alcohol extraction processes, soy protein concentrate (SPC) is made from defatted soy flour, resulting in a reduction of carbohydrate and some taste components [9]. SPC improves the functional and sensory quality of manufactured food systems through extrusion and proteolysis [10]. SPC obtained after acid or alcoholic extraction contains all amino acids, as well as 5% to 10% insoluble polysaccharides and less ash and can be utilized as a fortified ingredient for functional food development [11]. A study found that, in addition to several health benefits, SPC with genistein reduces kidney damage in nephrotic syndrome and cell proliferation [12]. Soy protein can help to lower blood and liver cholesterol levels, improve liver function in nonalcoholic steatohepatitis, and reduce insulin resistance [13, 14]. A study found that foods fortified with a mix of soy protein (soy grits and/or soy flour) and whole linseeds, such as bread and morning cereals, significantly improved plasma lipids in hypercholesterolemic patients [15]. Another study found that supplementing maize flour with soy flour dramatically improved nutritional status in children over the age of one year [16].

Although soy flour preparation has been the objective of some studies, none of them addressed the antioxidant properties of soy flours or their nutritional composition. Thus, we exhibited soy flours such as FF, DF, and SPC as a potential source of vegetable protein. In our study, we analyzed the nutritional composition of FF, DF, SPC, and raw soy flour. We also assessed the phytochemical content, and *in vitro* antioxidant activity of soy flours. This study has provided the essential data about nutritional quality and antioxidant activity of different states of soy flour to replace the synthetic antioxidant as well as create an opportunity to establish soy flour as a popular food product and ingredient around the world, including in Bangladesh.

## 2. Material and Methods

### 2.1. Sample Collection and Preparation

All samples were collected from the Institute of Food Science and Technology (IFST) and the Bangladesh Council of Scientific and Industrial Research (BCSIR), Dhanmondi, Dhaka, Bangladesh. After collecting, the samples were graded to ensure high grading quality of the samples.

### 2.2. Chemicals and Reagents

Methanol, hydrochloric acid, sodium hydroxide, aluminum chloride, sodium carbonate, sodium hydroxide, and potassium ferricyanide were purchased from Merck, Darmstadt, Germany. Ferric chloride, ascorbic acid, tannic acid, catechin, Folin-Ciocalteu reagent, and 2,2-diphenyl-1-picrylhydrazyl (DPPH) were purchased from Sigma Co. (St. Louis, Missouri, USA). Ascorbic acid and NBT were acquired from BDH Co. and ferrozine from Loba, India. The chemicals and reagents were of analytical grade.

### 2.3. Proximate Analysis

Moisture, ash, protein, fat, and crude fiber content was determined following the method suggested by the association of official analytical chemists (AOAC) [17, 18]. Moisture content was determined through evaporation weight loss at 105°C for 6 to 8 hours. Ash content was determined through a muffle furnace burning at 600°C for 6 hours before white ash. Protein content was determined by the Kjeldahl method using nitrogen to protein conversion factor 6.25 for soy flour [19]. Digestion, distillation, and titration were the three steps in the Kjeldahl method. Fat content was determined by extracting them in hexane, and after evaporating the hexane, residues left behind were determined. Crude fiber content was carried out by taking fat-free samples, boiling them with 200 ml (1.25%) sulfuric acid under reflux before further filtration, and washing with hot water to make the sample nonacidic. Then the residue was boiled again with 200 ml (1.25%) NaOH before filtration and washed with hot water to make the sample non alkaline. Then it was cooled and weighed. After incinerating in a muffle furnace at about 600°C for 20 minutes, we then cooled, weighed, and calculated the crude fiber. Carbohydrate content was estimated by subtracting the sum of ash, protein, fat, moisture, and crude fiber content [20]. Energy conversion factors, also known as Atwater factors [21], were used to calculate the energy content of the four products.

### 2.4. Minerals Determination

Minerals were analyzed following the methods described in the Manual of Laboratory Techniques of AOAC [18]. Weighed samples and ash were prepared in a muffle furnace at 600°C for 6 hours. The stock solution was prepared by using 6 M HCI, and the atomic absorption spectrophotometer (AAS, model: Thermo Scientific, ICE 3000 series, USA) was used for determining the mineral contents.

### 2.5. Amino Acid Analysis

The amino acid analysis was conducted following the method described by Malebana et al. [22] with slight modifications. The sample was prepared by acid hydrolysis with 6 N hydrochloric acid (HCl) at 110°C for 22 hours. Then the sample was filtrated with Whatman No. 1 filter paper and made the volume up to the mark with 0.1 N hydrochloric acid (HCl). Pentane was used to extract the amino acids, which were then separated using gradient elution on a chromatograph. The chromatograph was a Shimadzu Japan SpectraSystem P4000 Quaternary high-performance liquid chromatography with a SpectraSystem FL3000 fluorescence detector and a Rheodyne 7125 valve with a 20-liter injection loop. A concave curve was used to vary the eluents from sodium citrate buffer (pH 2.95)–acetonitrile (70 : 30) to sodium citrate buffer (pH 4.5)–methanol acetonitrile (14 : 6 : 70) at a flow rate of 1.4 ml/min. The amino acids were identified using a 264 nm excitation wavelength and a 340 nm emission wavelength. The analyzer showed the standard curve for the standard solution and another curve for the unknown sample solution. By comparing the areas of the two curves, amino acids were calculated.

### 2.6. Analysis of Phytochemicals and *In Vitro* Antioxidant Activity

Methanol extract and methanol to water extract were used for determining the antioxidant activity of SPC to compare with the antioxidant activity of raw soy flour, full-fat soy flour, and defatted soy flour.

#### 2.6.1. Preparation of Sample Extract

*(1) Methanol Extract*. Methanol extracts (ME) of four samples were prepared by dispersing them in 99.9% methanol. 1.0 g of each sample was dissolved in 10 ml methanol in a falcon tube with occasional shaking and stirring, finally sonication for 30 min [23]. This process was repeated five times to collect 50 ml of methanol extract from every specific sample (i.e., raw soy flour, full-fat soy flour, defatted soy flour, and soy protein concentrate). The supernatant ME was obtained by filtering the mixture through Whatman No. 1 filter paper and stored at 4°C until use.

*(2) Aqueous Extract*. An 80% methanol (purity 99.9%) plus 20% water (distill water) mixture solution was used for preparing the aqueous extract solution and storing it at 4°C until use.

#### 2.6.2. Determination of Total Phenolic Content

Total Phenolic Content (TPC) was estimated according to previously described methods [23, 24] with slight modification. 0.5 ml of extract (concentration of extract is 1.0 mg/ml) of the four samples and 0.5 ml of Folin-Ciocalteu reagent (0.5 N) were mixed individually and incubated at room temperature for 5 min. Then 2.0 ml saturated sodium carbonate was added and further incubated for 30 min at room temperature, and the absorbance was measured at 765 nm, and gallic acid (GA) [25] was used as a standard. The contents of total phenolics were extrapolated by using the linear equation *y* = 98.419*x* − 0.7443 (*R*^2^ = 0.9857), where *x* is the absorbance and *y* is the concentration of gallic acid, and *y* = 101.21*x* + 1.9659 (*R*^2^ = 0.9963) for tannin only for SPC as the second standard.

#### 2.6.3. Determination of Total Flavonoid Content

Total flavonoid content was determined by the aluminum chloride method [26] using rutin hydrate (RH) as a standard. 1 ml extract and 4 ml of water were added to a volumetric flask of each sample individually. After that, 0.3 ml of 5% sodium nitrite and 0.3 ml of 10% aluminum chloride were added and incubated at room temperature for 5 minutes. 2 ml of 1 M sodium hydroxide was added to the mixture, and the volume was made up to 10 ml with distilled water. The absorbance of the reaction mixture was measured at 510 nm against a blank spectrophotometrically (UV-VIS Specord 205). Total flavonoid content was calculated as (mcg/100 g) using the following equation based on the calibration curve: *y* = 638.95*x* + 9.8716, *R*^2^ = 0.9899, where *x* was the absorbance and *y* was the RH concentration.

#### 2.6.4. Determination of Total Antioxidant Capacity

The determination of total antioxidant activity was done using the phosphomolybdenum (Mo) method with slight modifications. The basic principle is based on the reduction of Mo (VI) to Mo (V) by the extract, and the subsequent formation of a green phosphate Mo (V) complex at an acidic pH. 0.5 ml of each sample's extract was mixed with 3.0 ml of reagent solution (0.6 M H_2_SO_4_, 28 mM Na_3_PO_4_, and 4 mM ammonium molybdate). The tubes containing the reaction solution were then capped and incubated at 95°C for 90 min. After the samples had cooled to room temperature, the absorbance of the solution was measured at 695 nm against a blank by a spectrophotometer. Methanol (0.3 ml) was used as the blank. The antioxidant activity is expressed as mg of the equivalent of gallic acid and as mg gm of the equivalent of vitamin C as the second standard for SPC.

#### 2.6.5. Determination of Reducing Power (RP)

The reducing power can be determined by the method described by Jayanthi and Lalitha [27] with some modifications. Various concentrations (0.2 ml, 0.4 ml, 0.6 ml, 0.8, ml, and 1.0 ml) of the methanol and aqueous extracts in an identical solvent were blended with phosphate buffer (2.5 ml) and potassium ferricyanide (2.5 ml). This mixture was cooled at 50°C for 20 min. Then 2.5 ml of 10% trichloroacetic acid was appended and centrifuged at 3000 rpm for 10 minutes. Distilled water (2.5 ml) and freshly prepared FeCl_3_ solutions (0.5 ml) were mixed and measured at 700 nm for absorbance by a spectrophotometer. Ascorbic acid was used as a standard.

#### 2.6.6. Determination of Ferric Reducing Power (FRAP)

The FRAP assay was carried out according to the method of Maizura et al. [28] with some modifications. FRAP reagent was prepared by dissolving 10 mM TPTZ solution in 40 mM HCL and 20 mM iron (III) chloride solution in a proportion of 10 : 1 : 1 (v/v) with acetate buffer. The reagent was prepared daily and stored at 370°C. At 593 nm in a UV visible spectrophotometer (UV-VIS 1200, Shimadzu Corporation, Japan), absorbance was measured. Ascorbic acid was used as the standard.

#### 2.6.7. DPPH Scavenging Activity

By using 2,2-diphenyl-1-picrylhydrazyl (DPPH), the free radical scavenging activity was determined by the previously described method [29] with some modifications. Take 0.2 ml, 0.4 ml, 0.6 ml, 0.8 ml, and 1.0 ml of methanol and aqueous extract in a test tube and made the final volume up to 1.0 ml with water. Then add 3.0 ml of DPPH stock (0.004%) solution and mix well. Incubated the mixture for 10 minutes in a dark place. Control was prepared from DPPH solution and methanol. Absorbance was measured by a spectrophotometer at 517 nm with methanol as a blank. The % of inhibition can be calculated by

(1) $$\begin{array}{c} \text{Inhibition }\left(\%\right)=\left(\frac{A_{0}–A_{1}}{A_{0}}\right)\times 100, \end{array}$$

where *A*_0_ is the absorbance of the control and *A*_1_ is the absorbance of the test.

The IC_50_ value is the quantity of antioxidants required to eliminate half of all free radicals in the body.

#### 2.6.8. Free Radical Scavenging Activity of ABTS

Free radical scavenging activity of ABTS was conducted according to the method used by Jiri et al. with some modifications [30]. 4.95 mmolL-1 potassium peroxo disulphated (*m* = 0.01338 g/10 ml) are mixed and dissolved in distilled water with seven mmolL-1 ABTS (*m* = 0.03841 g/10 ml). The solution was diluted in a 1 : 9 v/v ratio with distilled water. Incubated the solution mixture at dark for 12 hours and stored at 4°C temperature for up to 7 days. Fill different test tubes with 0.2 ml, 0.4 ml, 0.6 ml, 0.8 ml, and 1.0 ml of methanol and aqueous extract and made volume up to 1.0 ml with water. 3.0 ml ABTS reagent mixture was then added, properly mixed, and incubated at room temperature. After 5 min incubation, the absorbance was measured at 670 nm. Control was prepared with water and reagent. Gallic acid was used as a standard solution. The percentage of inhibition can be determined by the following equation and expressed as IC_50_,

(2) $$\begin{array}{c} \text{Inhibition }\left(\%\right)=\left(\frac{A_{0}–A_{1}}{A_{0}}\right)\times 100, \end{array}$$

where *A*_0_ is the absorbance of the control and *A*_1_ is the absorbance of the test.

#### 2.6.9. Statistical Analysis

All experiments were replicated three times. The mean and standard deviation were used to express the data. The R program (haven package) was used to determine the one-way ANOVA for group comparison and the Tukey's Honest Significant Difference (HSD) test for pair-wise comparison between different samples. A statistically significant level of probability was defined as ^∗^ is equal to *p* < 0.05, ^∗∗^ is equal to *p* < 0.01, ^∗∗∗^ is equal to *p* < 0.001, and ns is equal to nonsignificant.

## 3. Results

### 3.1. Proximate Analysis of Different States of Soy Products

**Figure 1:**
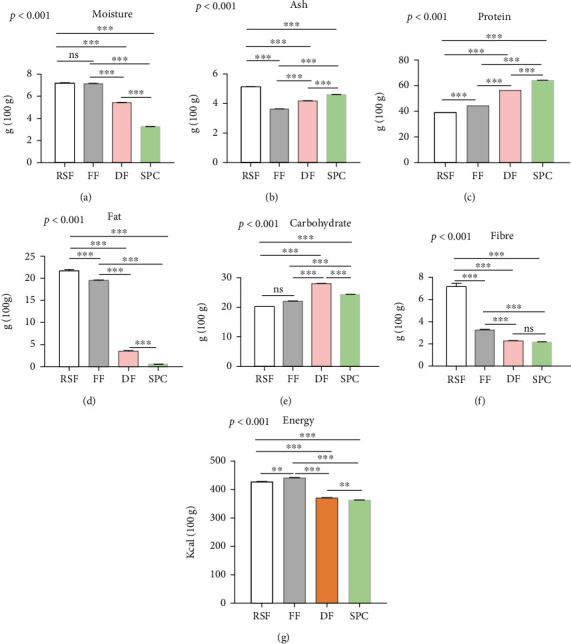
Proximate analysis of different soy products. The bar indicated the content of moisture (a), ash (b), protein (c), fat (d), carbohydrate (e), fiber (f), and energy (g). Data are shown as Mean ± SD for triplicate experiments. We applied the ANOVA test for multiple groups and pair-wise Tukey's Honest Significant Difference (HSD) test between two groups. *p* < 0.001 is the significant level of the ANOVA test and ^∗^ is p <0.05, ^∗∗^ is *p* < 0.01, ^∗∗∗^ is *p* < 0.001, and ns is nonsignificant for the significance level of Tukey's HSD test.

The proximate analysis includes the analysis of protein, fat, ash, moisture, fiber, and carbohydrate content of various states of soy flours. The comparison of nutrients of different states of soy flour is indicated in Figure 1. The results indicate that the moisture content is significantly (*p* < 0.001) different among various groups (Figure 1(a)). The results showed that the RSF and FF exhibited significantly (*p* < 0.001) higher moisture content (7.16% and 7.13%, respectively) compared to DF and SPC. On the other hand, SPC contained significantly (*p* < 0.001) lower moisture content compared to RSF, FF, and DF. Similar results were also found for the fat and fiber percentages of RSF, FF, DF, and SPC. RSF contains significantly (*p* < 0.001) higher fat and fiber content (21.17% and 7.15%, respectively) compared to others, and SPC contains significantly (*p* < 0.001) lower fat (0.54%) and fiber (2.15%) content compared to RSF, FF, and DF (Figures 1(d) and 1(f)). On the other hand, the protein percentages of four samples gradually increased from RSF to SPC (ANOVA, *p* < 0.001). The findings reported that SPC contains the highest protein percentages (65.14%) among the four groups, whereas RSF contains lower protein percentages (39.18%) compared to SPC, DF, and FF (Figure 1(c)). Furthermore, the study also revealed that the four samples also contained significantly (*p* < 0.001) different amounts of ash and carbohydrate (Figures 1(b) and 1(e)). Results indicate that RSF contains significantly (*p* < 0.001) fewer carbohydrates content compared to DF, FF, and SPC, and DF contains comparatively more carbohydrates than the others. Additionally, the energy content also significantly (ANOVA, *p* < 0.001) varies among the four groups. FF exhibited higher energy content (441.5 Kcal per 100 g), whereas SPC stood for lower energy content (362 Kcal per 100 g).

### 3.2. Micronutrients Analysis of Soy Products

**Figure 2:**
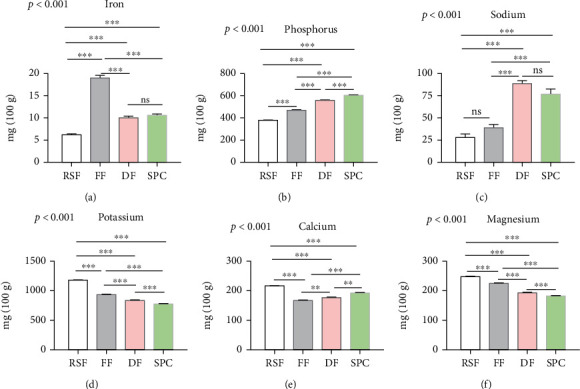
The content of minerals in soy products. The bar indicated the content of iron (a), phosphorus (b), sodium (c), potassium (d), calcium (e), and magnesium (f). Data are shown as *Mean* ± *SD* for triplicate experiments. We applied the ANOVA test for multiple groups and pair-wise Tukey's Honest Significant Difference (HSD) test between two groups. *p* < 0.001 is the significant level of the ANOVA test and ^∗^ is *p* < 0.05, ^∗∗^ is *p* < 0.01, ^∗∗∗^ is *p* < 0.001, and ns is nonsignificant for the significance level of Tukey's HSD test.

Micronutrients are essential components of food products. In our study, we analyzed the mineral contents (e.g., iron, phosphorus, sodium, potassium, calcium, and magnesium) to determine the micronutrient status of different soy products. Our results showed that the mineral contents (e.g., iron, phosphorus, sodium, potassium, calcium, and magnesium) of RSF, FF, DF, and SPC are significantly different (ANOVA, *p* < 0.001) for all groups. In the case of iron content, results showed that FF contained significantly (*p* < 0.001) higher iron content (19.013 mg/100 g) than RSF, DF, and SPC, and the RSF contained significantly (*p* < 0.001) less iron content (6.23 mg/100 g) compared to other soy products (Figure 2(a)). The study also found that the phosphorus content of soy products gradually increased from RSF to SPC, whereas SPC contains a significantly (*p* < 0.001) higher phosphorus content (603.24 mg/100 g) than other soy products (Figure 2(b)). Like iron, the sodium content (28.09 mg/100 g) in RSF is significantly (*p* < 0.001) lower than DF and SPC but no significant difference between RSF and FF (Figure 2(c)). Furthermore, the results also indicated that the potassium and magnesium contents are sharply decreased from RSF to SPC (Figures 2(d) and 2(f)). The results highlighted that the RSF contained significantly (*p* < 0.001) higher potassium (1178.6 mg/100 g), calcium (216.77 mg/100 g), and magnesium (247.55 mg/100 g) content compared to other soy products, and the SPC contains significantly lower potassium and magnesium content than RSF, FF, and DF (Figures 2(d), 2(e), and 2(f)).

### 3.3. Amino Acid Analysis of Soy Protein Concentrate

**Figure 3:**
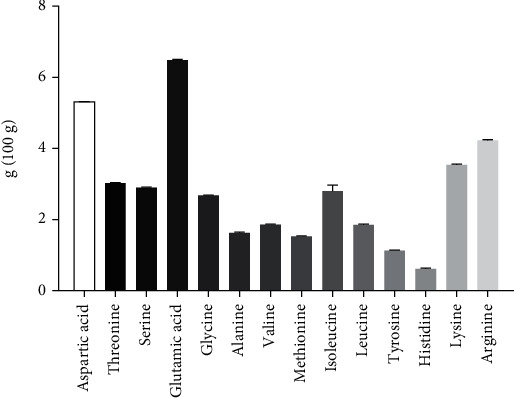
Amino acid content of soy protein concentrate. Data are shown as *Mean* ± *SEM* for triplicate g/100 g.

The amino acid profile denotes the protein quality of a food product. As we targeted developing soy protein concentrate from soy flour, we analyzed the amino acid profile of SPC. In this study, the amino acid content was expressed in percentage on the dry basis of the product (Figure 3). The result demonstrated that the SPC contained most of the essential amino acids except some. The percentages of glutamic acid (6.49%), aspartic acid (5.31%), and arginine (4.2%) are significantly higher in SPC compared to other amino acids. On the other hand, the percentages of histidine (0.62%), tyrosine (1.13%), and methionine (1.53%) are significantly (*p* < 0.05) lower in SPC compared to other amino acids. In contrast, the essential amino acids phenylalanine and tryptophan are unable to be detected in our analyzed product, as well as the nonessential amino acids proline and cysteine. However, it lacks might not be a result of its absence in the raw material but rather of its disintegration as a result of the processing and acid hydrolysis of the sample that took place during its preparation for amino acid analysis. Altogether, results suggest that the protein percentage is higher in SPC but may lacks some amino acids in the analyzed product.

### 3.4. Comparative Phenolics and Flavonoids Content in Soy Products

**Figure 4:**
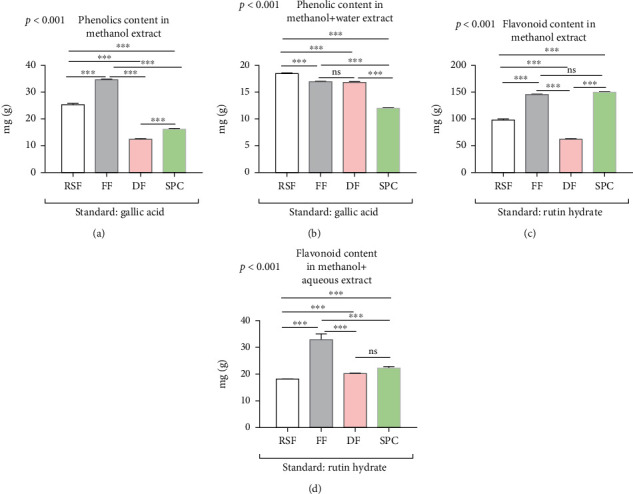
Phenolics and flavonoids analysis of different soy products. The bar indicated the content of phenolics in methanol extract (a), phenolics in methanol and aqueous extract (b), flavonoids in methanol extract (c), and flavonoids in methanol and aqueous extract (d). Data are shown as *Mean* ± *SD* for triplicate experiments. We applied the ANOVA test for multiple groups and pair-wise Tukey's Honest Significant Difference (HSD) test between two groups. *p* < 0.001 is the significant level of the ANOVA test and ^∗^ is *p* < 0.05, ^∗∗^ is *p* < 0.01, ^∗∗∗^ is *p* < 0.001, and ns is nonsignificant for the significance level of Tukey's HSD test.

Like other nutrients, we have analyzed the phenolic and flavonoid content of soy products. In this case, we extracted the sample by using both methanolic and aqueous methanolic solvents. The results showed that the phenolic and flavonoid content of soy products (RSF, FF, DF, and SPC) differed significantly (ANOVA, *p* < 0.001) in both solvent groups (Figure 4). The results also showed that the phenolic content is significantly (*p* < 0.001) higher in FF (34.73 mg/g of extract) compared to RSF, DF, and SPC in methanol extract equivalent to gallic acid (Figure 4(a)). On the other hand, the RSF contains significantly (*p* < 0.001) higher phenolic content (18.12 mg/g extract) than other aqueous methanolic extracts, equivalent to the gallic acid standard (Figure 4(b)). Notably, the findings demonstrated that the overall phenolic content in soy products is significantly (ANOVA, *p* < 0.001) higher in methanol extract compared to aqueous methanolic extract.

On the other hand, in methanolic extract, the flavonoid content is significantly (*p* < 0.001) higher in SPC (149.67 mg/g extract) compared to other soy products but significantly (*p* < 0.001) decreased in aqueous methanolic extract equivalent to the rutin hydrate standard (Figures 4(c) and 4(d)). Furthermore, the DF and RSF contain significantly (*p* < 0.001) lower flavonoid content (61.97 mg and 18.18 mg/g of extract) in methanolic and aqueous methanolic extract, respectively. More importantly, the methanolic extract of all soy products showed significantly (*p* < 0.001) higher flavonoid content compared to aqueous methanolic extract (Figures 4(c) and 4(d)). These findings suggest that the phenolic and flavonoid content of soy products varies, and also depends on different solvents extraction methods.

### 3.5. Total Antioxidant Analysis of Soy Products

**Figure 5:**
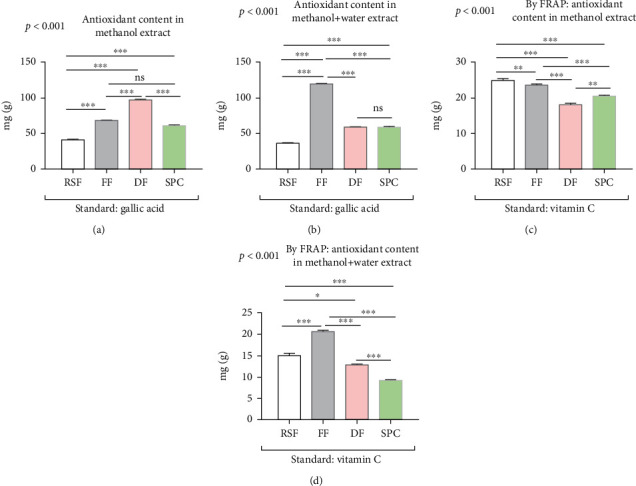
Total antioxidant analysis of different soy products. The bar indicated the content of total antioxidant content in methanol extract by phosphomolybdenum blue method (a), total antioxidant content in the aqueous methanolic extract by phosphomolybdenum blue method (b), antioxidant content in methanol extract by FRAP method (c), and antioxidant content in the aqueous methanolic extract by FRAP method (d). Data are shown as *Mean* ± *SD* for triplicate experiments. We applied the ANOVA test for multiple groups and pair-wise Tukey's Honest Significant Difference (HSD) test between two groups. *p* < 0.001 is the significant level of the ANOVA test and ^∗^ is *p* < 0.05, ^∗∗^ is *p* < 0.01, ^∗∗∗^ is *p* < 0.001, and ns is nonsignificant for the significance level of Tukey's HSD test.

The total antioxidant content of RSF, FF, DF, and SPC was determined by phosphomolybdenum blue methods against a gallic acid standard and expressed as milligrams per gram (mg/g) of extract. The results found that the total antioxidant content of RSF, FF, DF, and SPC were significantly different (ANOVA, *p* < 0.001) from each other in both methanolic and aqueous methanolic groups (Figure 5). The results demonstrated that DF contains significantly (*p* < 0.001) higher total antioxidant (97.59 mg) compared to other soy products, whereas RSF contains (41.45 mg) lower total antioxidant in the methanolic extract group (Figure 5(a)). Similarly, in the aqueous methanolic extract, the total antioxidant content is significantly (*p* < 0.001) lower in RSF (35.20 mg) but dramatically increased in FF (119.42 mg). On the other hand, the total antioxidant content of SPC almost remains constant in both extracts (Figures 5(a) and 5(b)).

Furthermore, we also used the ferric reducing antioxidant power (FRAP) method for analyzing the total antioxidant content of soy products against vitamin C as a standard. The results showed that RSF and DF contain significantly (*p* < 0.001) higher antioxidants (25.19 mg/g and 20.72 mg/g, respectively) in methanolic and aqueous methanolic extracts, respectively. On the other hand, DF and SPC contain significantly (*p* < 0.001) lower antioxidant content (18.29 mg/g and 9.26 mg/g) in methanolic and aqueous methanolic extracts, respectively (Figures 5(c) and 5(d)). Additionally, in aqueous methanolic extract, the antioxidant content of all soy products dramatically decreased compared to methanolic extract groups.

### 3.6. Free Radical Scavenging Capacity and Reducing Power of Soy Products

#### 3.6.1. ABTS Scavenging Activity

**Figure 6:**
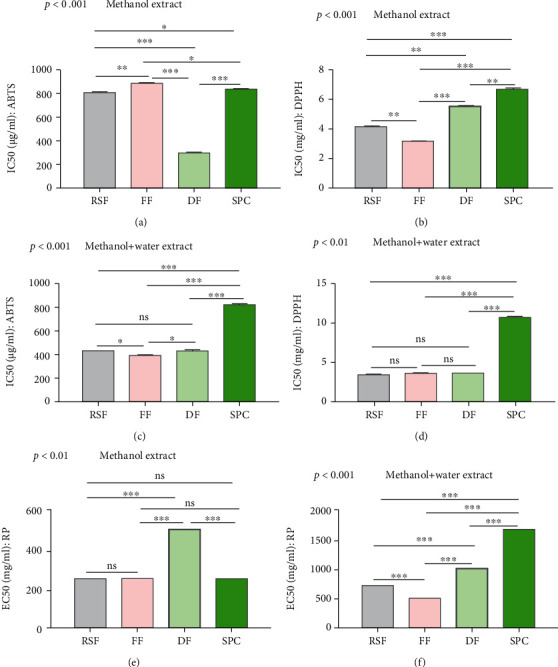
Free radical scavenging capacity and reducing power of soy products. The bar indicated the IC_50_ of ABTS assay in methanol extract (a), IC_50_ of ABTS assay in aqueous methanolic extract (b), IC_50_ of DPPH assay in methanol extract (c), IC_50_ of DPPH assay in aqueous methanolic extract (d), EC_50_ of reducing power assay in methanol extract (e), and EC50 of reducing power assay in aqueous methanolic extract (f). Data are shown as *Mean* ± *SD* for triplicate experiments. We applied the ANOVA test for multiple groups and pair-wise Tukey's Honest Significant Difference (HSD) test between two groups. *p* < 0.001 is the significant level of the ANOVA test and ^∗^ is *p* < 0.05, ^∗∗^ is *p* < 0.01, ^∗∗∗^ is *p* < 0.001, and ns is nonsignificant for the significance level of Tukey's HSD test.

We investigated the *in vitro* free radical scavenging activity of antioxidants found in soy products after determining the overall antioxidant content of soy products. For both the methanolic and aqueous extracts, the 50% scavenging activity, i.e., IC_50_ (mg/ml), values were calculated to determine their ability to inhibit the ABTS radicals. The lower the IC_50_, the higher the free radical scavenging power. Our experiment results found that soy product extract showed free radical scavenging power in ABTS methods against gallic acid standards (Figures 6(a) and 6(c)). In the methanolic extract, the DF showed a significantly (*p* < 0.001) lower IC_50_ (299.61 *μ*g fresh product/ml) than RSF, FF, and SPC. On the other hand, the FF showed a significantly (*p* < 0.05) higher IC_50_ (887.01 *μ*g fresh product/ml) compared to others. A higher IC_50_ denotes lower free radical scavenging power. In contrast, the IC_50_ of FF is significantly (*p* < 0.05) lower in aqueous methanolic extract, whereas SPC showed a higher IC_50_ in the same extract. These findings suggest that soy products may be able to remove free radicals in the ABTS model.

#### 3.6.2. DPPH Scavenging Activity of Soy Products

DPPH is one of the greatest antioxidant processes for determining the scavenging activity of radicals. The analysis results showed that the FF exhibited significantly (*p* < 0.01) lower IC_50_ (3.16 mg fresh product/ml) in methanolic extract and SPC showed significantly (*p* < 0.001) higher IC_50_ (6.67 mg fresh products/ml) in the same extract against ascorbic acid standard (Figure 6(b)). In the case of aqueous extracts of RSF, FF, DF, and SPC, the IC_50_ values were 3.54 mg, 3.71 mg, 3.68 mg, and 10.81 mg fresh product/ml, respectively (Figure 6(d)). Ascorbic acid's IC_50_ value was 9.75 *μ*g/ml. IC_50_ values of DF and SPC are significantly (*p* < 0.01, *p* < 0.001) higher than RSF in methanol extracts. The IC_50_ values of RSF, FF, and DF values are significantly (*p* < 0.001) lower than the SPC IC_50_ values in aqueous extract. These findings suggest that soy products may have the potential to reduce free radicals in the DPPH model.

#### 3.6.3. Analysis of Reducing Power (RP) of Soy Products

In the case of reducing power (RP), ascorbic acid was used as the reference compound, and it indicates the transformation of ferric (III) from ferrous (II) form. The RP was identified to be higher with increasing concentration (0.2 ml to 1.0 ml). The experiment results showed that the EC_50_ values of RSF, FF, DF, and SPC in methanol extracts were 249.53 mg/ml, 250.78 mg/ml, 499.79 mg/ml, and 250.0 mg/ml, respectively (Figure 6(e)), where EC_50_ refers to the half maximal effective concentration for reducing free radical by the extract. The product's increased antioxidant activity is indicated by the lower EC_50_ value. In aqueous extracts, EC_50_ values were 714.39 mg of fresh product/ml, 500.08 mg of fresh product/ml, 1000.09 mg of fresh product/ml, and 1665.94 mg of fresh product/ml for RSF, FF, DF, and SPC, respectively (Figure 6(f)). The EC_50_ value for standard ascorbic acid was 3.047 mg/ml. The EC_50_ value of SPC is significantly (*p* < 0.001) lower than that of DF in methanol extract. Interestingly, in aqueous extract, the SPC EC_50_ value is significantly (*p* < 0.001) higher than RSF, FF, and DF. These findings suggest that the antioxidants present in soy products may have the potential to remove half of the free radicals in an *in vitro* model.

## 4. Discussion

The commercial importance of soy products is notably due to their high nutrients and good qualities of protein. In this study, we analyzed the macro- and micronutrient and *in vitro* antioxidant activity of different soy products. The analysis results revealed that the nutrient content significantly (ANOVA, *p* < 0.001) varies from each other. The proximate analysis described that the SPC contains the highest protein percentages and lower fat and moisture percentages compared to other soy products. Interestingly, the protein percentage of SPC in our study was significantly consistent with the previous study [31]. The SPC contains low fat due to the removal of fat during the preparation of SPC. On the other hand, the moisture, fat, and fiber content are significantly (*p* < 0.001) higher in RSF than in others (Table 1). This may happen due to the processing of raw soy flour from soy protein concentrate. Additionally, a previous study found that SPC contains 0.5 to 1.0% of fat, and interestingly, this is consistent with our findings [32]. Studies reported that the high protein content of SPC has many beneficial effects on health for instance reducing chronic inflammatory diseases, protecting gut epithelial function, and lower low-density lipoprotein levels (LDL) [33–35].

On the other hand, the micronutrient analysis revealed that soy products contain a significant amount of minerals in a different state of product processing. Notably, the results reported that the phosphorus content of soy products significantly increased after the removal of fat, and potassium and magnesium significantly decreased (Table 1). However, the previous study showed that soy products contained about 4.5 to 13.35 mg sodium, 23.35 to 624.45 mg potassium, 8.9 to 61.35 mg calcium, and 33.35 to 142.60 mg phosphorus per 100 g [36]. Interestingly, our finding found higher mineral content than previous studies, and this may happen due to different methods of soy flour processing and preparation. The high protein and minerals content of soy products may contribute to the human health. For example, the role of iron, calcium, and phosphorus in bone and teeth development is strongly recognized [37]. Additionally, the mineral helps to maintain neuromuscular activity, fetal growth, and healthy immune function [38]. Our analysis results reported that soy products contain a significant amount of minerals that could help the body with various mechanisms. In terms of amino acid content, the soy products contain essential amino acids but lack some essential amino acids, and this section needs further validation for reporting the protein quality of soy products. However, the lack of these two amino acids may be due to disintegration as a result of the processing and acid hydrolysis that occurred on the sample during its preparation for amino acid analysis in the HPLC system, rather than their absence in the raw material. Previous study reported that tryptophan is not detected in soybean products [39], and that is consistent with our findings. Similarly, we were unable to detect phenylalanine in our product by using our methods. Although, research reported that soy product contains essential amino acids, but sulphur containing amino acid is considered limiting amino acids in soybean [40, 41].

Apart from the macro- and micronutrient analysis in soy products, we determined the phytochemicals and antioxidant content of RSF, FF, DF, and SPC. The results illustrated that the phenolic content of soy products is 25.39, 34.73, 12.41, and 16.18 mg/g, respectively, in methanol extract and 18.12, 16.55, 16.45, and 11.85 mg/g, respectively, in aqueous extract equivalent to gallic acid standard. Additionally, results also reported that flavonoid content of soy products is 97.16, 145.26, 63.69, and 150.47 mg/g, respectively, in methanolic extract and 18.32, 34.19, 20.24, and 22.88 mg/g, respectively, in aqueous extract equivalent to gallic acid standard. As our findings, a previous study described that soybean and soybean products could be a potential source of phenolics and flavonoids. The study revealed that the phenolic content of various soybeans ranged from 1.15 to 1.77 mg per g equivalent to the gallic acid standard. Furthermore, the study also described that the total flavonoid content of soybean and soy products ranging from 0.68 to 2.13 mg per g equivalent to the quercetin standard [42]. Another study reported that the phenolic content of soybean ranges from 10.3 to 13.7 mg/g per extract [43]. In addition, the study revealed that soybean contains 6.4-81.7 mg/g of phenolics equivalent to gallic acid, flavonoids 3.5-44.6 mg/g equivalent to quercetin [44]. We detected more phenolics and flavonoid content in this study than in prior studies; however, this could be due to differences in sample preparation, analysis methodology, and analysis standard. More importantly, studies described that the phenolics, flavonoids, and antioxidants content of soybean could be varied from variety to variety, and also depends on the environment of cultivation [44, 45]. Furthermore, methanol extract had higher flavonoid and phenolic content than water extract, indicating that methanol is an excellent solvent for phytochemical extraction.

In addition, we analyzed the total antioxidant content and analyzed their free radical scavenging power in various *in vitro* models. The analysis results revealed that four soy products contain significantly higher antioxidant content and free radical scavenging power in various *in vitro* models. The antioxidant content of soy products ranges from 18.29 to 25.19 mg per gram equivalent to gallic acid standard. On the other hand, a previous study described that soybean and soy products are a potential source of antioxidants and phytochemicals [46]. In this study, the results showed *in vitro* antioxidant activity by DPPH, ABTS methods, and reducing power assay. In DPPH methods, the IC_50_ of soy products was 3.54 to 10.81 mg per ml of fresh product where ascorbic acid was used as a standard. A previous study reported that the antioxidant from soybean or soy products showed free radical scavenging power in DPPH methods, and IC_50_ was 0.14 to 0.80 mg per ml of fresh product equivalent to the ascorbic acid standard [44]. Lower IC_50_ indicates a better inhibition capacity of free radicals. The IC_50_ is lower in our study and it may vary due to the standard we used and the different varieties of soybean we used. On the other hand, the IC_50_ of soy products in ABTS methods was 299.61 to 887.1 *μ*g per g of products equivalent to the gallic acid standard. Furthermore, in reducing power assay, the EC_50_ of the methanolic and aqueous extract is significantly different between various products. In the methanolic extract, DF shows higher EC_50_ than other products. On the other hand, the EC_50_ of RSF, FF, and SPC is not significantly (*p* > 0.05) different from each other. Lower EC_50_ represents higher antioxidant activity against free radicals and higher EC_50_ indicates lower antioxidant activity against free radicals. In the case of aqueous extract, FF shows significantly (*p* < 0.001) lower EC_50_ compared to others, and EC_50_ is significantly higher in SPC. Altogether, these findings summarized that FF in aqueous extract shows better antioxidant activity compared to other products. Interestingly, a previous study also described the *in vitro* antioxidant activity of soybean and soy products and also found that antioxidants from soybean can reduce free radicals in ABTS methods equivalent to Trolox [47]. Additionally, another study also found that the IC_50_ of soybean was 77.9 mg and 5.5 mg per gram extract equivalent to gallic acid standard in DPPH and ABTS methods, respectively [48]. These findings are also consistent with our findings and suggested that antioxidants from soybean and soybean products could be a good source of antioxidants and remove free radicals in our bodies. Notably, the soy antioxidant not only reduces free radical but also plays an important role in various health benefits such as lower cholesterol from fat, preventing heart diseases, and reducing the risk of diabetics [49]. Continue to support this, according to a study, soy protein products can lower blood total cholesterol, low-density lipoproteins (LDLs), and triglycerides when consumed instead of animal protein. Apart from their lipid-lowering properties, fermented soy products have also been shown to help reduce the effects of diabetes, high blood pressure, heart disease, and cancer [13]. More crucially, the study found that incorporating soy flour as a dietary ingredient boosted the protein level as well as other nutrients in the food and prevented bone loss in women who consumed it [50]. Another study illustrated that soy flour addition to bread significantly increased the physicochemical, nutritional, and sensorial properties of functional beef burgers [51]. Furthermore, the study also described that soy protein concentrate significantly changes the dough characteristics and gluten properties [52]. However, our results demonstrated a lack of some essential amino acids in our analyzed products, and this section needs further analysis. Based on the findings, we may conclude that soy products (flour) contain high protein, minerals, phytochemicals, and antioxidants, which could be adopted as a potential food ingredient for functional food development. The health benefits of the soy products discussed may be validated by further *in vivo* or clinical research.

## 5. Conclusion

Finally, the findings showed that the nutritional and phytochemical content of the soy product varied depending on the state. Nutrient analysis results showed that the SPC contains significantly (*p* < 0.001) higher protein and lower fat content than RSF, FF, and DF. On the other hand, the RSF contains lower protein content and more fat than others. Micronutrient analysis showed that the content of micronutrients significantly (*p* < 0.001) varies from state to state in soy products. The RSF contains higher potassium, calcium, and magnesium content and significantly (*p* < 0.01) changes during processing. The iron and phosphorus content are significantly (*p* < 0.001) higher in FF and SPC, respectively, compared to other products. In comparison to gallic acid, rutin hydrate, and vitamin C standard, the results demonstrated that all soy products contain a significant (*p* < 0.001) amount of phenolics, flavonoids, and antioxidants, respectively. Additionally, *in vitro* free radical scavenging power analysis by ABTS and DPPH assays showed that both methanolic and aqueous extracts of soy products significantly (*p* < 0.001) reduce the free radicals against gallic acid and ascorbic acid standards, respectively. Altogether, these results suggest that soy products (flours) especially DF and SPC could be a potential ingredient for developing protein, minerals, and phytochemical-rich plant-based functional foods.

## Figures and Tables

**Table 1:** Macro- and micronutrients composition of different state of soya protein.

Parameter	Nutrients	RSF (% ± SD)	FF (% ± SD)	DF (% ± SD)	SPC (% ± SD)
Proximate Analysis	Moisture	7.16 ± 0.015	7.13 ± .035	5.42 ± 0.038	3.26 ± 0.012
	Ash	5.13 ± 0.009	3.62 ± 0.014	4.17 ± 0.015	4.61 ± 0.012
	Fat	21.17 ± 0.29	19.50 ± 0.11	3.55 ± 0.08	0.54 ± 0.023
	Protein	39.18 ± 0.015	44.4 ± 0.008	56.6 ± 0.009	65.14 ± 0.015
	Fibre	7.15 ± 0.29	3.24 ± 0.069	2.27 ± 0.02	2.15 ± 0.04
	Carbohydrate	20.22 ± 0.026	22.09 ± 0.06	28 ± 0.062	24.29 ± 0.058

Minerals		**Mg/100** g	**Mg/100 g**	**Mg/100 g**	**Mg/100 g**
	Iron	6.23 ± 0.18	19.01 ± 0.59	10.05 ± 0.38	10.62 ± 0.29
	Phosphorus	379.78 ± 3.52	468.26 ± 5.13	559.17 ± 4.48	603.24 ± 4.55
	Potassium	1178.6 ± 8.45	932.79 ± 2.08	835.26 ± 4.4	778.37 ± 2.8
	Sodium	28.09 ± 3.89	38.84 ± 3.85	88.57 ± 3.59	76.82 ± 5.73
	Calcium	216.77 ± 0.83	167.36 ± 0.63	176.57 ± 0.58	192.74 ± 0.45
	Magnesium	247.55 ± 1.32	225.12 ± 0.7	193.19 ± 1.54	182.37 ± 0.65

## Data Availability

The data used to support the study's findings are supplied in the paper.
